# Polymeric Nanofibers for Drug Delivery Applications: A Recent Review

**DOI:** 10.1007/s10856-022-06700-4

**Published:** 2022-12-03

**Authors:** Xiaoge Duan, Hai-lan Chen, Chunxian Guo

**Affiliations:** 1 College of Animal Science and Technology, Guangxi University, Nanning, 530005 China; 2 School of Materials Science and Engineering, Suzhou University of Science and Technology, Suzhou, 215009 China

## Abstract

With the rapid development of biomaterials and biotechnologies, various functional materials-based drug delivery systems (DDS) are developed to overcome the limitations of traditional drug release formulations, such as uncontrollable drug concentration in target organs/tissues and unavoidable adverse reactions. Polymer nanofibers exhibit promising characteristics including easy preparation, adjustable features of wettability and elasticity, tailored surface and interface properties, and surface-to-volume ratio, and are used to develop new DDS. Different kinds of drugs can be incorporated into the polymer nanofibers. Additionally, their release kinetics can be modulated via the preparation components, component proportions, and preparation processes, enabling their applications in several fields. A timely and comprehensive summary of polymeric nanofibers for DDS is thus highly needed. This review first describes the common methods for polymer nanofiber fabrication, followed by introducing controlled techniques for drug loading into and release from polymer nanofibers. Thus, the applications of polymer nanofibers in drug delivery were summarized, particularly focusing on the relation between the physiochemical properties of polymeric nanofibers and their DDS performance. It is ended by listing future perspectives.

**Figure:**
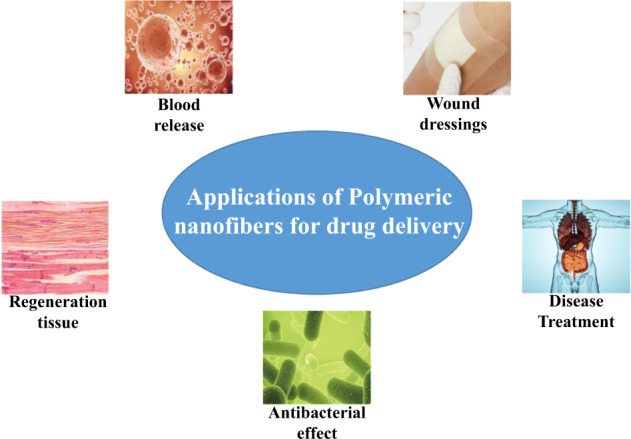
Graphical abstract

Graphical abstract

## Introduction

Drugs play an indispensable role in diagnosing, preventing, and treating human and animal diseases. In recent decades, the number of drugs consumed each year is increasing worldside, due to the development of the economy, increase of life expectancy, growth of the human population, increase of work and life pressure, changes in the living environment, and increased awareness of health care, et al. According to the statistics, global drug consumption in 2016 was US $1153.1 billion, and this number increased to US $1298.7 billion in 2020, with 74.3% for chemical drugs, 20.8% for biological agents, and 4.8% for peptides. Among these drugs, antineoplastic drugs account for 40%, cardiovascular drugs account for 30%, antiviral drugs account for 20% and autoimmune disease drugs account for 10% [1]. As a result, drugs have become an integral part of people’s and animals’ lives. A DDS is a formulation or device that introduces a drug into the body and delivers it to a specific target organ or tissue while controlling the rate and duration of drug release in the body to improve efficacy and safety.

Current drug delivery methods include oral cavity (such as tablets, capsules, and granules), injectable delivery (such as intravenous, intramuscular, and dermal), respiratory delivery, and transdermal delivery. For the oral route of drug administration, most drugs will pass through the organs of the mouth, esophagus, stomach, and intestines and be absorbed into the body’s circulation in the small intestine to exert their effects, which has the greatest advantage of convenience. Oral drugs are usually less expensive and convenient to treat, but not easily absorbed large molecules, such as proteins, and the bioavailability of the drug is affected by factors such as physicochemical and biological barriers [2, 3]. Injection administration can be divided into intramuscular, subcutaneous, and intravenous injections, mainly the drug from the injection site into the capillary circulation/vein, and then to the body circulation to exert drug effects [4, 5]. Injectable drug delivery is characterized by rapid drug absorption, rapid increase in blood concentration, and rapid therapeutic effect, but there may be local pain and risk of infection, and it is not suitable for the delivery of suspensions, oil solutions, and those containing coagulants. Respiratory delivery is generally used for pulmonary administration and refers to the administration of drugs in the form of aerosols, sprays, or very small solid particles, where the large surface area and abundant capillaries of the lungs provide advantages for drug absorption [6]. Transdermal drug delivery is to apply the drug to the skin surface and penetrate the subcutaneous tissues through structures such as skin follicles, ducts, or microchannels, which are absorbed through capillaries to exert drug effects [7]. Transdermal delivery avoids hepatic metabolism, protection from gastrointestinal effects, continuous drug delivery, and good patient compliance [8]. In summary, choosing the appropriate route of administration could lead to better drug efficacy.

Most traditional drugs are immediate-release formulations, which exhibit drawbacks of drug degradation in the gastrointestinal tract, uncontrollable drug concentration in target organs/tissues, excessive accumulation in non-target tissues/organs, quick clearance, unavoidable adverse reactions, and so on [9, 10]. For example, when using chemotherapeutics to kill cancer cells, the bone marrow system, digestive system, immune system, and other important systems are also seriously damaged, resulting in vomiting, anemia, infections, loss of hair, and other clinical symptoms [11, 12]. Diabetes is a disease affecting more than 537 million individuals and the only way to control the blood glucose level is to use insulin [13]. However, due to the quick clearance of insulin, individuals need to intramuscular inject insulin before every dine, inducing pain in the patients and producing lots of medical waste. The way that how drugs are administrated has a great impact on pharmacokinetics, distribution, pharmacodynamics, metabolism, and thus their therapeutic effect and toxicity. An ideal drug delivery system (DDS) is one in which drugs can be delivered to a target organ/tissue or cells at a controllable rate and minimizes unwanted side effects [14, 15].

With the development of nanotechnologies and nanomaterials, new DDS using nanoparticles, nanofibers, hydrogels, and microspheres are developed to overcome the limitations of traditionally immediate release formulations [16–18]. For example, extended-release agents are used to reduce the number of doses administered [19, 20], targeted drug delivery is developed to reduce drug toxicity [21, 22], and enteric solvents are adopted to mitigate the effects of the gastric environment on drug action [23, 24], and controlled-release systems are produced to enhance targeting and accuracy [25]. At the same time, nanofibers have a small diameter of 1-100 nm and have a large porosity and high specific surface area, so they have a wide range of applications in major fields [14, 26, 27]. Due to their composition, nanofibers are generally classified into three types: polymeric nanofibers, inorganic nanofibers, and organic/inorganic composite nanofibers, among which polymer nanofibers are often used as carriers and widely used in drug delivery due to their high biocompatibility and stability, high specific surface area and volume ratio, high porosity and high similarity to extracellular matrix (ECM) [14]. Currently, polymeric nanofibers can be loaded with proteins [28], polysaccharides [29], and growth factors [30], and it has also been used to load lipophilic drugs such as ibuprofen and paclitaxel [31, 32] and hydrophilic drugs such as ciprofloxacin hydrochloride and metronidazole [33, 34]. A timely and comprehensive summary of polymeric nanofibers for DDS is thus highly needed but still lacking.

In this review, we first describe the common methods for polymer nanofiber preparation, followed by introducing the controlled techniques for drug loading into and release from polymer nanofibers. Thus, the applications of polymer nanofibers in drug delivery were summarized, particularly focusing on the relation between the physiochemical properties of polymeric nanofibers and their DDS performance. This review is ended by providing conclusions and listing future perspectives.

## Preparation methods of polymer nanofibers

**Fig. 1:**
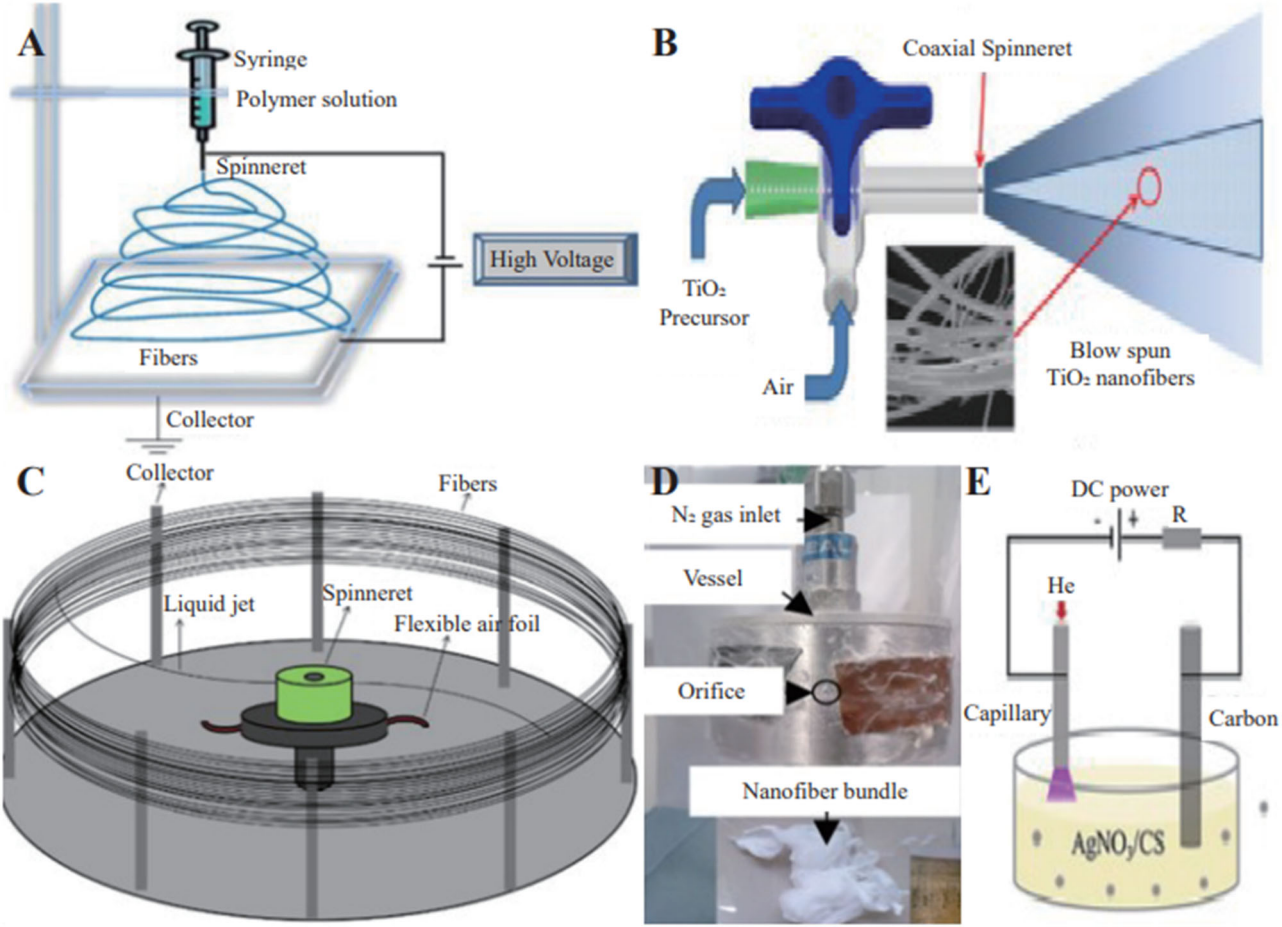
Common preparation methods of polymeric nanofibers. electrospinning system (**A**) [149]; Pressurized gyration system (**B**) [150]; centrifugal spinning system (**C**) [151]; pressurized gyration system (**D**) [69]; plasma-induced synthesis system (**E**) [76]

Polymer nanofibers are generally made of two types of polymers, natural and synthetic [35–38], by melt-blown method [39], template synthesis [40], self-assembly method [41], direct stretching method [42], wet spinning method [43], electrostatic spinning method, centrifugal jet spinning method, plasma-induced synthesis, pressurized spinning, solution blowing spinning [44], and other methods. Most of these techniques, however, have certain drawbacks which limit their wide applications. For example, the melt blowing is limited to thermoplastic polymers [45], the nanofibers prepared by the template synthesis method are short and it is difficult to remove the nanofibers from the template [46], and the direct-drawing technique is difficult to produce fibers with a diameter of less than 100 nm, and the production speed is slow [47], while the materials required for self-assembly preparation are usually amphiphilic and costly to produce [41]. In contrast, electrospinning, centrifugal jet spinning, plasma-induced synthesis, pressure spinning, and solution blow spinning are compatible with various materials and the fabrication process is very simple. Figure 1 summarizes the common methods for polymer nanofibers fabrication.

### Electrospinning

In terms of drug delivery, diverse polymers, whether natural or synthetic, require electrospinning to generate fibrous scaffolds that imitate ECM [48–50]. Due to the huge surface area to volume ratio of electrospinning nanofibers, it is the most extensively utilized approach for preparing polymer nanofibers [51]. In 1934, A. Formhals et al. proposed a patent for electrospinning cellulose derivatives (such as cellulose acetate) to make rayon [52]. The principle of electrospinning technology is that a high voltage electrostatic field transports the polymer solution to the spinning needle, where it is stretched and polished by the electric field force to produce nanofibers at the collection end. Basic electrospinning, hybrid electrospinning, emulsion electrospinning, melt electrospinning, coaxial electrospinning, and triaxial electrospinning are six types of electrospinning procedures that utilize polymers as raw materials [38].

Zhang et al. [53] used chitosan and polyvinyl alcohol electrospun nanofibers mats. Also, different levels of bioactive components from the Chinese herbal medicine dandelion were loaded into the electrospinning solution to create composite nanofiber mats to investigate the wound dressing properties of composite nanofiber materials and their ability to treat diabetic wounds. In addition, Allafchian et al. [54] prepared 3D nanofiber scaffolds containing aloe vera, polyvinyl alcohol, and tetracycline hydrochloride by electrostatic spinning method for cell culture applications. However, when employing electrospinning technology to get uniform fibers, it must be equipped with a strong electric field, the production efficiency is low, and it is difficult to combine into a large-scale 3D network, all of which limit the technology’s popularity and application [55–57].

### Solution blow spinning

Medeiros et al. [58] were the first to disclose employing pressured gas to generate nanofibers from polymer solutions in 2009. Due to its lack of an electric field, great production efficiency, and low cost, solution jet spinning technology has attracted the attention of academics both at home and abroad [56]. Air-jet spinning is another name for solution blow spinning. The procedure is separated into four steps: construction of the cone, drawing of the jet, coagulation of the jet, and fiber deposition [59]. The inner cavity contains the polymer solution, while the outer cavity contains the pressured gas. The polymer solution is expelled from the top of the cone and stretched towards the stationary collector due to pressure drop and shear at the gas/solution interface caused by the pressurized high-velocity gas. As the solvent evaporates, the pulled polymer stream quickly produces fibers. However, the solvents used in the preparation process may be toxic, costly to produce, and not available for mass production. Zhou et al. [60] reported a solution blowing method and cold pressing procedure for nanofibers composite films of polyvinylidene fluoride/polyaniline at ambient temperature, laying the groundwork for the industrialization of energy storage devices. In addition, Silva et al. [61] investigated the application of nanofiber membranes as fruit ripening monitoring sensors using poly (lactic acid)/poly (aniline) solution blow spinning.

### Centrifugal spinning

Centrifugal spinning, also known as centrifugal spinning or rotary jet spinning, employs centrifugal force to overcome the surface tension of a polymer solution and spin it into a variety of fibers with diameters ranging from microns to nanometers [62, 63]. The centrifugal spinning machine composes of a collector, a spinneret, a shaft, and a motor. The nanofiber preparation procedure begins with the insertion of polymer solution into the spinneret and is followed by a high-speed rotation. The polymer solution is ejected from the nozzle of the spinneret and stretched by centrifugal force. Finally, polymer nanofibers are obtained when the polymer solution is solidified on the collector [64]. The centrifugal spinning production process does not require high pressure and is more productive than other methods. Therefore, it is suitable for commercial production. However, the highly rotating bearings spinning will generate a large load, which will produce shaft bearing fracture phenomenon, affecting production efficiency and raising costs. Lu et al. [65] prepared superabsorbent sodium alginate/polyethylene oxide submicron fibers containing the hydrophilic model drug tetracycline hydrochloride by centrifugal spinning.

In addition, the discovery research of nozzle less centrifugal spinning provides a solution for the clogging of the nozzle of the spinneret in conventional centrifugal spinning, which can be done by adding spin coaters [66], rotating spinning discs with flow controllers [67], or using interchangeable spinnerets [68]. Chen et al. [67] prepared polyethylene terephthalate fibers and polyvinyl pyrrolidone fibers into nanofiber membranes by melt-needle-free centrifugal spinning technology. The preparation process begins with the injection of polymer liquid from the liquid controller to the inner wall of the disk and the formation of a film, and then the condensation of the film produces multiple jets, which are sprayed from the edge of the disk and finally sprayed onto the collector.

### Pressurized gyration

Pressurized gyration, also known as core-sheath pressurized spinning, was first described by Mahalingam and his colleagues in 2013 according to the Rayleigh-Taylor instability principle of spinning polymer solutions [69–71]. The core area was made of polycaprolactone, and the sheath area was made of polyvinyl alcohol and hydroxyapatite nanocrystals to prepare core-sheath fiber scaffolds. Pressurized gyration is more capable of mass production than electrospinning techniques. The most important advantage is that no electric field is required, and the solvent is not limited by solubility or vapor pressure. Cam et al. [72] prepared polylactic acid nanofibers patches loaded with progesterone by electrospinning and pressurized spinning. The experimental results show that compared with the pressure rotation, the size of the patch obtained by electrospinning is smaller and the dispersion performance is better.  However, it is worth mentioning that the yield of nanofibers prepared by pressurized rotation is higher than that of electrostatic spinning. Therefore, pressurized rotation is more suitable for the large-scale production of nanofibers.

In addition, to confirm that the composite nanoclay polymer fibers can be fabricated as scaffolds for bone tissue engineering applications, Kundu et al. [73] designed a pressurized rotating device to combine polycaprolactone fibers with urea hydroxyapatite and montmorillonite nanoclay to prepare a 3D bracket. The test results show that the polymeric fiber scaffold has good biocompatibility and can increase the content of alkaline phosphatase in the body, thereby promoting the differentiation of bone marrow mesenchymal stem cells, and can be used as a material for preparing non-healing scaffolds for bone tissue regeneration.

### Plasma-induced synthesis

Plasma-induced synthesis uses electricity to create plasma, which allows precursor atom nanostructures to expand into fibers over time. The electrode is made of the precursor material, which is immersed in an electrolyte solution and subjected to an electric current to create a plasma in the solution. The atoms form clusters as the plasma expands and the discharge time rises, which are oxidized and developed into fibers [74, 75]. Meanwhile, Nanofibers prepared by plasma-induced synthesis exhibit certain antibacterial properties. Sun et al. [76] developed in situ synthesis of antibacterial silver nanoparticles/chitosan (AgNP/CS) nanocomposites with effective antibacterial properties against Escherichia coli and Staphylococcus aureus strains. Agrawal et al. [77] studied the use of plasma-induced synthesis technique and TiO_2_ nanoparticles of 15–20 μm to enhance the biocompatibility of polymethyl methacrylate, and prepared a 20 μm polymer nanocomposite film by solution casting, further using oxygen ions Plasma for membrane modification. However, the incident ions used in plasma-induced synthesis may cause damage to biomolecules in cells and tissues and require high current systems such as vacuum, atmospheric pressure devices, and high energy supply, which may reduce the cost efficiency of the process [75].

## Drug loading strategies of the polymer nanofibers based DDS

**Fig. 2:**
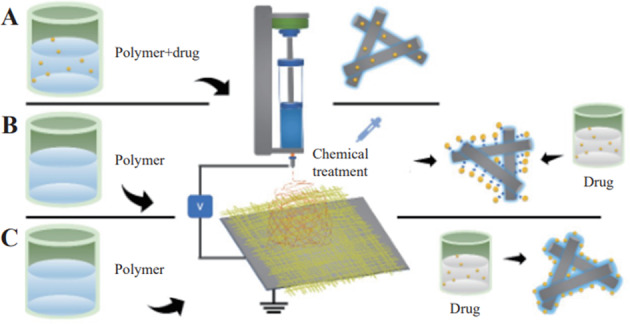
Schematic diagram of different drug loading methods, such as encapsulation (**A**), chemical immobilization (**B**), and physical adsorption (**C**) [152]

Drug loading technology is a crucial stage in achieving the optimal release mechanism, and it is influenced by a variety of elements such as the drug’s solubility, the combination with the material, and so on. According to data, hydrophobic compounds account for around 60% of all drugs on the market, and organic solvents are frequently used for the dissolution of them [78]. Figure 2 shows the schematic diagram of different drug loading methods. And Table 1 lists the advantages and disadvantages of each method.

**Table 1:** Advantages and disadvantages of each drug loading method

Methods	Advantages	Disadvantages
Encapsulation	• Larger amount of fixed medication • Drug activity is not easily altered • The easy and affordable methods • Increased solubility • Reduces degradation during drug delivery	• Finiteness of pore size • Potential to reduce the biocatalytic rate
Chemical immobilization	• Strong binding between drug and nanofiber carrie • Easy to quantify • Multiple binding modes	• Modification of the chemical structure leads to a change in the functional conformation
Physical adsorption	• Nanofiber carriers are reusable if the drug is deactivated • Simple and inexpensive method	• The adsorption effect is not strong and may be dislodged during the release process

### Encapsulation

Encapsulation is a method of encapsulating a drug in a polymer, and the encapsulation process can load more hydrophilic or hydrophobic drugs due to the high specific surface area and volume ratio of nanofibers, and the entire drug delivery system can avoid degradation of the active drug component and deliver the drug in a directed or continuous manner, thus offering advantages such as increased drug solubility, reduced drug degradation and improved drug bioavailability [79]. However, the diffusion limitations imposed by the nature of the carrier that encapsulates the drug in a polymer, which in some cases may reduce the biocatalytic rate [80], is a drawback of the current polymer encapsulation of drugs. Currently, drug encapsulation is possible only with biodegradable and biocompatible polymers such as some natural polymers (chitosan, cellulose, etc.) [81, 82] and synthetic polymers (polycaprolactone, polyvinyl acetate, etc.) [83, 84].

**Fig. 3:**
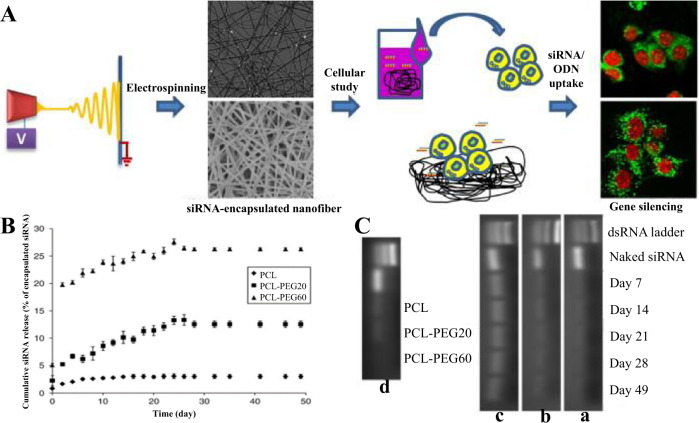
Flow chart of siRNA experiments encapsulated in polycaprolactone nanofibers (**A**), In vitro release test results (**B**), and analysis of siRNA integrity in polymeric nanofibers (**C**) [85]

Cao et al. [85] achieved the controlled release of siRNA by encapsulating siRNA in polycaprolactone nanofibers. Figure 3A shows siRNA encapsulated in polycaprolactone nanofibers by electrostatic spinning technique and subjected to morphological characterization, in vitro release assays, and other tests. FESEM images show that the average fiber diameters of pure PCL, PCL-PEG20 (20 mg/mL), and PCL-PEG60 (60 mg/mL) had mean fiber diameters of 309.4 ± 7.0 nm, 335.0 ± 7.0 nm, and 423.5 ± 9.8 nm, respectively. Figure 3B shows the in vitro release of siRNA for 49 days and detects that pure PCL, PCL-PEG20 (20 mg/mL), and PCL-PEG60 (60 mg/mL) were consistently released for at least 28 days and the cumulative release was 3.01, 12.55, and 26.30%, respectively, indicating that the addition of PEG significantly enhanced the release of siRNA. The integrity of the siRNA in the composite nanofibers was also analyzed, and Fig. 3C shows that the siRNA maintained some integrity even after the electrostatic spinning process. And the integrity was examined on its 7th, 14th, 21st, 28^th^, and 49th days during the release process, and the results are shown in Fig. 3C in the a–c graphs showing that the molecular weight of siRNA recovered from the supernatant is consistent with the molecular weight of bare siRNA and maintains the integrity for at least 49 days, and d graph shows that after 49 days of siRNA release, the siRNA samples extracted from the nanofiber scaffold gel electrophoresis results, slight siRNA bands were observed, probably due to factors such as low concentration and limited extraction efficiency. In addition, Basar et al. [86] made an emulsion with the anti-inflammatory drug ketoprofen in the oily phase and gelatin dissolved in acidified water in the watery phase. The electrospinning technique was used to convert the stable oil-in-water (O/W) emulsion into a polycaprolactone/gelatin nanofibers membrane, which was then fixed with glutaraldehyde. The polycaprolactone/gelatin nanofiber membrane demonstrated sustained drug release capabilities for up to 4 days when compared to the single polycaprolactone nanofiber membrane. In addition, the cell test revealed that the ketoprofen-containing nanofibers membrane had no detrimental side effects on cells.

### Chemical immobilization

Another method of immobilizing the drug is through covalent bonding. Covalent bonding between drug and carrier is a method to enhance the performance of slow and controlled release of drug by covalent bonding between drug and carrier [87]. The advantage of covalent bonding is that it can improve the binding and stability between composite nanofibers, and also the method of immobilization by covalent bonding facilitates better observation of drug loading and plays a quantitative role. Mateo et al. [88] also found that for multi-point binding of bioactive substances to the carrier surface, the method of covalent bonding immobilization may be the best. However, chemical modifications such as the use of cross-linking agents during the preparation process may lead to changes in the functional conformation of the drug and the carrier, causing certain effects.

**Fig. 4:**
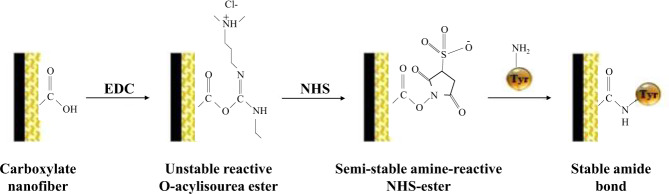
Schematic representation of tyrosinase immobilized in nanofiber mats by EDC/NHS activation [90]

Choi et al. [89] electrospun amine-terminated polyethylene glycol with polycaprolactone to expose the amine group, and acted nerve growth factor (NGF) on mesenchymal stem cells at the same time to complete investigations on NGF release. In another study, tyrosinase is extremely versatile in the market and is often used to catalyze a variety of reactions, Dagli et al. [90] prepared polyacrylonitrile/polyurethane/m-aminobenzoic acid nanofibers by electrostatic spinning and immobilized tyrosinase by EDC-NHS activation. Figure 4 shows the process of tyrosinase immobilization in nanofiber mats by EDC/NHS activation. The results showed the average diameters of PAN/PU and PAN/PU/P3ANA nanofibers containing 0.075, 0.150, and 0.300 mg P3ANA, respectively, were 103 ± 11, 144 ± 24, 111 ± 17, and 119 ± 22 nm. The amount of immobilized tyrosinase determined by the BCA method showed that about 87% of the tyrosinase was covalently bound to the nanofibers.

### Physical adsorption

In addition to using encapsulation and covalent bonding methods, researchers often use physisorption to achieve binding between the drug and the carrier. Many factors influence physisorption, including the interaction of hydrophobic and hydrophilic forces, van der Waals forces, etc. Currently, physisorption immobilization using van der Waals forces is one of the simplest immobilization methods and the advantage of a high specific surface area to volume ratio of polymeric nanofibers can increase the drug loading capacity [91]. However, the weak binding of the drug to the carrier in physisorption may cause drug shedding. Siqueira et al. [92] studied the design of PLA/chitosan nanofibers for the adsorptive immobilization of lipase and after two applications, the immobilized enzyme activity was significantly reduced. Thus physical adsorption methods are less frequently applied for drug delivery in cancer therapy.

**Fig. 5:**
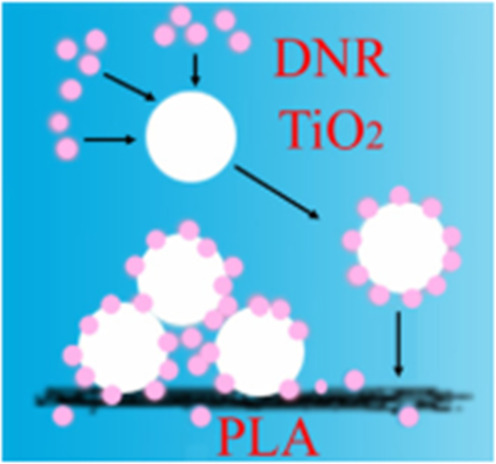
Schematic diagram of the self-assembly of roxithromycin on TiO_2_-PLA nanofibers [93]

Chen et al. [93] investigated the production of nanofiber materials from polylactic acid (PLA) as a starting material, combining the anticancer drug daunorubicin with TiO_2_ particles on the nanofibers. Daunorubicin is positively charged throughout the overall system, while TiO_2_ particles and polylactic acid are negatively charged, and due to non-covalent interaction, they produce composite nanomaterials that are utilized to analyze the release of the anticancer drug daunorubicin. Figure 5 shows a possible schematic diagram of the loading of erythromycin on TiO_2_-PLA nanofibers. In addition, Ma et al. [94] developed chitosan/poly (ethylene oxide) nanofiber based on electrostatic interactions and used it to load paclitaxel to study the application of composite nanofibers in prostate cancer chemotherapy.

## Drug release methods of the polymer nanofibers-based DDS

**Fig. 6:**
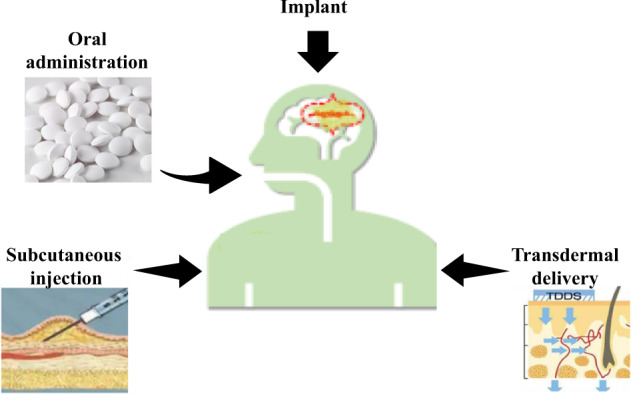
Various routes of drug administration

Researchers can choose the best drug delivery route based on factors including the drug’s site of action, mode of action, and bodily degradation, for example, the use of enteric solvents to overcome the impairment of drugs in the stomach and the use of extended-release agents to overcome the higher number of doses administered. Figure 6 depicts various drug release routes from polymeric nanofibers.

### Oral administration

Oral administration is currently the clinically main technique of administration, with oral pharmaceuticals accounting for more than half of all FDA-approved drugs [95]. The medicine is absorbed into the body through the digestive system, disseminated into the blood circulation by the drug molecule, and carried to the target tissue/organ to exert its medical action following oral delivery [96]. Oral administration contains the advantages of low price, convenience, easy and stable transportation, portability, and no direct damage to the skin. However, oral administration is to be absorbed through the mucous membrane of the gastrointestinal tract, drugs that are easily degraded by the gastrointestinal tract are not suitable, such as penicillin, insulin, etc., and are easily destroyed by oral administration and can only be used by injection.

For smart oral medication administration, Liang et al. [97] developed a self-ablative nanoparticle. They inserted the hemagglutinin-2 peptide into polylactic-glycolic acid nanoparticles modified with zwitterionic di-lauroyl phosphatidylcholine. In addition, Limoee et al. [98] used electrospinning technology to create polyvinyl alcohol (PVA)/carboxymethyl cellulose (CMC) nanofibers that were then loaded with the drug pramipexole to create a new oral drug delivery system for the treatment of Parkinson’s disease. In another study, Akhgari et al. [99] prepared folic acid enteric microfibers containing the pH-sensitive polymer Eudragit® S 100 by electrostatic spinning to overcome the sensitive environment caused by gastric acid and enzymes and to prepare suitable enteric reagent systems.

### Subcutaneous injection

Subcutaneous injection is usually a slow absorption of the drug through the subcutaneous extracellular matrix and into the bloodstream through the permeation of the endothelium [100]. Subcutaneous injections are generally indicated for drugs that cannot be administered via the gastrointestinal tract. It can also be used for local anesthesia or preoperative drug supply. The bioavailability of drugs delivered subcutaneously is said to be higher than that of drugs administered orally [101]. Furthermore, compared to oral delivery, subcutaneous injection results in faster, better absorption and higher blood drug levels [102]. However, subcutaneous administration is generally not an option when administered at high concentrations and may reduce serum levels [103].

Neuberg et al. [104] used photopolymerization to prepare polydiacetylene nanofibers, loaded siRNA cells, inhibited the oncogene Lim-1 in renal cancer cells, and confirmed the delivery of siRNA into subcutaneous tumors via polydiacetylene nanofibers via intraperitoneal injection, resulting in a novel system for delivering siRNA. In addition, Johnson et al. [105] successfully prepared various porous nanofibers made of poly (ε-caprolactone), poly (lactic-ethanolic acid), gelatin, gelatin methacrylate, bioglass, and magnetically responsive polymer composites. Compared to nonporous nanofibers, porous nanofibers are easier to grow human nerve cells because more neurons and a larger number of cells can be grown. In addition, after subcutaneous injection into rats, porous nanofibers have better biocompatibility than nonporous nanofibers.

### Implant

The implant is a drug formulation with a controlled release that is implanted subcutaneously or in other specific areas using a particular cannula or surgical process. Subcutaneous implants, as opposed to transdermal and oral controlled release formulations, penetrate the skin barrier and enable long-term drug release under the skin, avoiding first-pass effects and gastrointestinal enzymatic degradation while enhancing drug bioavailability. Implant applications range from contraceptive treatment to long-term or targeted drug delivery in multiple therapeutic areas, such as the cosmetic industry, but implant delivery systems may require secondary surgery to remove the implant.

Elshazly et al. [106] used a low-temperature sol-gel process to make bioactive glass, then combined it with a polymer solution and electrospun the glass sol to make nanofibers. The nanocomposites were implanted into the buccal folds of the maxillary mucosa of New Zealand male rabbits with type I diabetes to see if they might be employed as bioscaffolds for diabetics with weakened immune systems. In addition, the biocompatibility of titanium implants is not ideal in biomedicine, and the addition of polymeric nanofibers remedies this deficiency. Nhlapo et al. [107] summarized this year’s narrative of polymeric nanofibers loaded with titanium implants, in which Jahanmard et al. [108] electrostatically spun polycaprolactone and poly(lactic acid-ethanolic acid) nanofibers loaded with vancomycin and rifampicin onto titanium implants to study their controlled independent drug delivery systems and bactericidal effects.

### Transdermal delivery

The transdermal drug delivery is to coat the drug on the skin’s surface and penetrate the subcutaneous tissue via structures like hair follicles, conduits, or microchannels in the skin so that the drug can be absorbed by the capillaries in the subcutaneous tissue and transported to the entire body via blood systemic circulation [7]. Transdermal delivery, as opposed to typical oral and subcutaneous injections, avoids the liver’s first-pass action and is less invasive, painless, and cost-effective [109]. Drug encapsulation and release affect transdermal drug delivery, which is currently used in the treatment of numerous skin illnesses such as psoriasis, contact dermatitis, and skin cancer [110].

Transdermal nanocarrier systems can be divided into two modes of release: sustained release and activated modulated release. Shekh et al. [111] synthesized polymer nanofibers from polyacrylonitrile, which were then chemically modified with oxidized chitosan and loaded with acyclovir for drug release tests. Activated modulated release, unlike sustained release, necessitates a specific physical or chemical response, as well as a specified reaction state. For the activated modulated release, at present, there have been studies on temperature-responsive nanofibers [112, 113], and photothermal nanofibers [114, 115]. Zheng et al. [116] released the drug in response to temperature changes. They made temperature-responsive polymer nanofibers with olive oil as the core and Nisopropylacrylamide and N-methylol acrylamide (5:1) as raw ingredients using the coaxial electrospinning method. Meanwhile, the nanofiber carrier technology has a lot of promise for precise delivery. The transdermal method, unlike intravenous treatment, avoids the circulatory system, resulting in a lack of substantial permeability and retention effects. As a result, advances in nanofiber carriers are still required.

## Applications of polymer nanofibers-based DDS

**Fig. 7:**
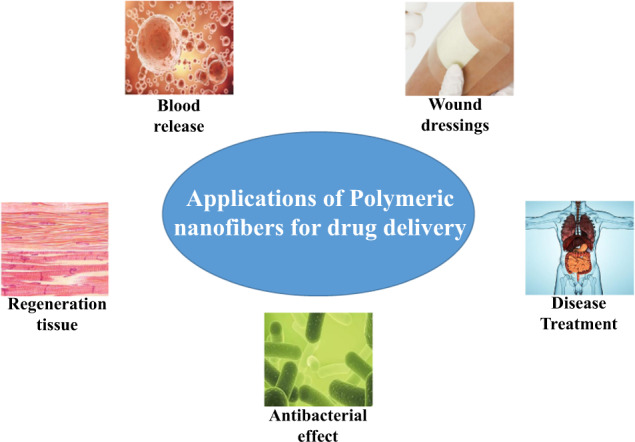
Summary diagram of polymeric nanofibers for drug delivery applications

Polymeric nanofibers are gaining more and more attention in many fields, especially in drug delivery, which can be used in several applications. Figure 7 schematically shows a schematic diagram of polymeric nanofibers in drug delivery applications.

### Regeneration tissue

The natural extracellular matrix (ECM) consists of various protein protofibrils and fibers interwoven in glycosaminoglycans (GAG) [117], and the fibrous scaffold formed by ECM protectively supports cells at all times. Meanwhile, nanofibers with ECM-like structures generated from natural and synthetic polymers are investigated for use in a variety of applications. For example, Niu et al. [118] used hyaluronic acid-functionalized collagen nanofibers in modulating macrophages to promote healing in urothelial regeneration. At present, nanofibrous structures have been found to significantly improve the use of tissue scaffold materials for bone, cartilage, cardiovascular, nerve, and bladder regeneration and reduce scar formation [119, 120], and the preparation of more complex intracorporeal scaffolds using composite nanofibers materials is becoming a reality, but the construction of scaffolds at the cell-matrix level has not yet been observed.

Rezk et al. [121] employed composite nanofibers made of polycaprolactone (PCL) and polyglycerol sebacate (PGS) as well as loaded hydroxyapatite nanoparticles (HANPs) and simvastatin (SIM) to mimic the bone extracellular matrix (ECM) to improve bone cell proliferation and regeneration processes. At the same time, the morphology, drug release, and cytocompatibility of the fiber mat were studied. The results showed that the average fiber diameters of PCL-PGS, PCL-PGS-HA, and PCL-PGS-HA-SIM reached 0.86 ± 0.34, 0.87 ± 0.35, and 0.88 ± 0.25 μm, respectively. The initial release of the PCL-PGS-HA-SIM nanofiber mat was around 20%, and it was gradually released slowly and virtually linearly until 24 h and released 79.5% within 7 days, according to the in vitro release study. The drug release data were consistent with the Korsmeyer–Peppas model and the Kopcha models. The growth of MC3T3E1 osteoblasts sown on various composite nanofiber mats was used to perform a cytocompatibility test. After day 2, different nanofibers samples showed similar cell adhesion and spreading results, while after day 6, the morphology of MC3T3E1 cells seeded on PCL-PGS-HA-SIM composite nanofibers confirmed that there are more associated fully extended cell layers and an increased rate of cell proliferation. Thus, this experiment provides the basis for bone tissue regeneration.

In another study, to enhance the fibroblast lineage differentiation of bone marrow mesenchymal stem cells, Xu et al. [122] used coaxial electrospinning technology to prepare silk fibroin/polylactic acid-caprolactone-polyethylene oxide into core-shell fibers for delivery of Fibroblast growth factor 2 and connective tissue growth factor. The test conditions were optimized, and the optimal concentration in the preparation process was determined to be 7.2 wv.% of silk fibroin in the core solution and 19.2 wv.% in the shell solution of PLA-caprolactone and polyethylene oxide at a concentration of 4.8 wv.%. The SEM image shows that the diameter of the prepared nanofibers is about 1.19 ± 0.34 µm. The in vitro release test shows that the initial burst release is 37.6 ± 1.8% within the first 8 h, and the cumulative release reaches 81.7 ± 1.8% on the 7th day. After day 7, the release profile showed a decelerating release of less than 1% per day, and on day 14 the cumulative release from the shell reached 91.6 ± 1.8%. In addition, to confirm that the composite nanoclay polymer fibers can be fabricated as scaffolds for bone tissue engineering applications, Kundu et al. [73] designed a pressured rotating device that included polycaprolactone fibers with urea hydroxyapatite and montmorillonite nanoclay to prepare a 3D bracket. The results show that the polymeric fiber scaffold has good biocompatibility and can raise alkaline phosphatase levels in the body, thereby promoting the differentiation of bone marrow mesenchymal stem cells. It may also be utilized to make non-healing scaffolds for bone tissue regeneration.

### Wound dressings

With a total thickness of 1.5–4.0 mm, the skin is the greatest organ required for life and functions as a barrier between the inside and outside of the body [123]. Although the skin has a natural ability to heal itself, open wounds are frequently infected by microorganisms that cause infection at the wound site and spread to nearby healthy tissues, delaying wound healing [124]. Wound dressing is one of the clinical therapeutic materials, but there is no ideal wound dressing that can meet all of the requirements of wound dressing. The requirements that a functional wound dressing should meet in clinical practice are (1) good breathability; (2) absorption of excess tissue exudate; (3) efficient protection of wounds from microbial infection; (4) promotion of tissue regeneration; (5) stronger hemostasis; (6) non-adherence to wounds; (7) provision of a moist environment; (8) non-toxic, biocompatible and easily degradable [125–127]. In recent years, polymeric nanofibers have gotten a lot of interest because of their high porosity and specific area to volume ratio, among other things. High porosity can promote cellular respiration, and a large specific surface area to volume ratio can activate cellular signaling pathways quickly [128]. Most importantly, polymer nanofibers have a structural shape that is remarkably similar to that of the natural extracellular matrix, allowing them to protect supporting cells and encourage cell proliferation while also healing damaged tissues [129].

**Fig. 8:**
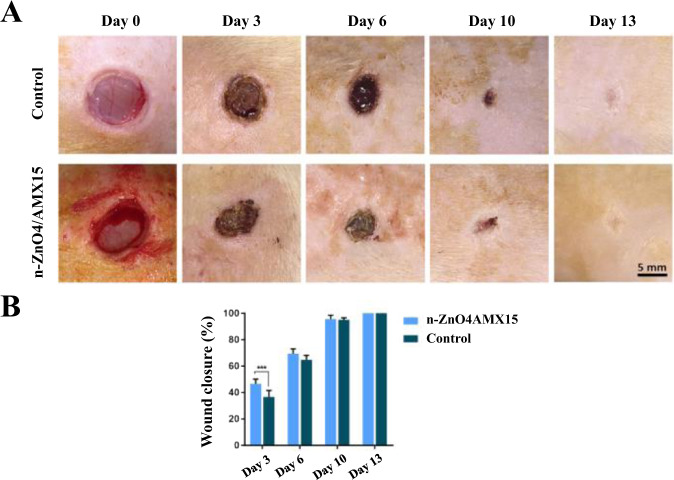
Optical images (scale bar = 5 mm) and wound shrinkage images of the wound site at different times in the control and experimental groups (***p < 0.001) [131]

Choi et al. [130] electrospun a mixture of PCL–PEG and PCL block copolymers into nanofibers sheets, submerged them in an aqueous solution, exposed functional amine groups on the nanofiber surface, and immobilized recombinant human epidermal growth factor (EGF) on electrospun nanofibers. EGF nanofibers increased keratinocytes expression and stimulated epidermal growth in human primary keratinocytes, according to the findings. In addition, Jafari et al. [131] made a bilayer nanofibrous scaffold with a top layer loaded with amoxicillin (AMX) and a bottom layer loaded with ZnO nanoparticles using polycaprolactone (PCL) and gelatin as raw ingredients. The average diameter of the constructed nanofibrous scaffolds was 576.36 ± 197.77 nm, according to SEM images. In vitro release assays showed that the materials had a rapid release time of 24 h and a slow release time of 144 h for amoxicillin, and paper diffusion and cytotoxicity testing validated the inhibition of bacterial growth and promotion of cell proliferation. Finally, in vivo tests on twelve male Spraguedawley rats (200–250 g) showed that the prepared nanofibers accelerated wound contraction and increased collagen deposition and angiogenesis, and in Fig. 8, A shows the optical images (scale bar = 5 mm) of the wound sites of the control and experimental groups (containing 4% ZnO and 15% AMX) at different time points, and B shows their corresponding wound contraction rate images. The images show that on day 3, the wound shrinkage was 36.73 ± 4.93 and 46.58 ± 3.66% in the control and experimental groups, respectively, but on day 6, the wound shrinkage was 64.77 ± 3.35 and 69.44 ± 3.65% in the control and experimental groups, respectively, and on day 10, the wound shrinkage was 95.07 ± 1.51, 95.60 ± 2.99%, and both reached healing levels on day 13, and the experimental group demonstrated a considerable healing effect in the first three days.

### Blood release

The advantages of polymeric nanofibers, such as large surface area and high porosity, have attracted much attention, and this advantage can enhance the interaction between cells and nanofibers, facilitating the preparation of novel materials for cell and blood release [132]. Blood release requires materials with anticoagulant properties, and polymeric nanofibers can be used for loading antithrombotic drugs to capture erythrocytes and complete blood release, such as heparin, nattokinase, aspirin [133]. It is also possible to prepare nanofibers with hydrophilic surface and other routes [134]. However, they currently exist only in the laboratory, and relevant animal experiments still need to be perfected.

**Fig. 9:**
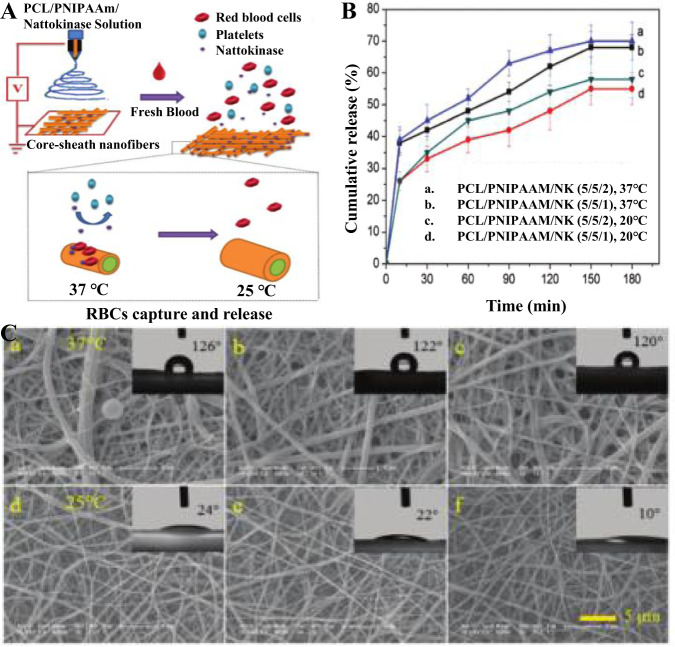
Schematic diagram of the procedure for the preparation of PCL/PNIPAAm nucleus-sheath smart nanofibers containing NK (**A**). The release profile of NK from PCL/PNIPAM/NK nanofibers (**B**). Measurement of adhesion and water contact angle of purified platelets on nanofibers (**C**). Where a, d for PCL/PNIPAAm nanofibers; b, e for PCL/PNIPAAm/NK (5/5/1) nanofibers; c, f for PCL/PNIPAAm/NK (5/5/2) nanofibers. a–c for temperature 32 °C, d-f for temperature 25 °C [136]

Shi et al. [135] prepared polycaprolactone/poly(N-isopropyl acrylamide) (PCL/PNIPAAm) nanofibers by electrostatic spinning with a single spinneret to capture and release erythrocytes by coupling bovine serum albumin to poly(N-isopropyl acrylamide) nanofibers, and then generated chemically cross-linked nanofiber platforms by thiene reactions. The prepared nanofiber platform was experimentally confirmed to be thermally responsive and hydrophilic-hydrophobic interchangeable, and capable of directly capturing red blood cells. At the same time, the captured erythrocytes were easily released in response to temperature stimulation, achieving a capture and release efficiency of up to 100%. In another experiment, Shi et al. [136] mixed poly-N-isopropylacrylamide (PNIPAAm), polycaprolactone (PCL) and nattokinase (NK) solutions in the ratio of 5/5/1 and 5/5/2, respectively, and prepared nanofibers with PCL/PNIPAAm core-shell layer by electrostatic spinning, Fig. 9A shows the smart PCL/PNIPAAm composite nanofibers loaded with NK Fig. 9A shows the schematic diagram of the preparation of NK-loaded smart PCL/PNIPAAm composite nanofiber. The in vitro NK release test showed that curves a and c in Fig. 9B showed higher cumulative NK release at a temperature of 37 °C, and curves a and b indicated that the higher NK loading resulted in higher release, and both composites could release NK for more than 180 min. After the measurement of water contact angle, it was found that PCL/PNIPAAm nanofibers could switch between hydrophobic and hydrophilic by temperature adjustment. a-c in Fig. 9C were all 37 °C and all were hydrophobic (water contact angle >120°), and when the temperature was lowered to 25°, as in Fig. 9C, d-f all showed hydrophilic (water contact angle <24°), and, as the higher NK loading, more tends to be hydrophilic, which may be due to the predominant hydrogen bonding between PNIPAAm and water molecules at a temperature of 25 °C. Upon heating, the intramolecular hydrogen bonding of PNIPAAm replaces the intermolecular hydrogen bonding, which leads to the hydrophobicity of the nanofibers. In summary, when the nanofibers come in contact with blood, NK is released from the nanofibers to facilitate the capture of red blood cells (RBCs) from the blood, and the captured RBCs are released in a nondestructive manner due to temperature changes, obtaining a release efficiency of up to 100%. When the temperature is about 32 °C, NK is released from the nanofibers to facilitate the capture of RBCs as indicated by the nanofibers, and when the temperature is below 32 °C, the nanofibers complete the hydrophobic-hydrophilic switch to facilitate the release of RBCs without damage.

### Antibacterial effect

Nanofibers have a high specific surface area and volume ratio, allowing oxygen to pass through, while submicron-sized pores have been found to “filter” bacteria [137]. At the same time, in order to better improve the antibacterial performance, there are several ways. Firstly, the nanofibers themselves are supplemented with other antimicrobial materials, for example, supramolecular assemblies using polyethylene glycol-b-polylysine (PEG-b-PLL) and ethylenediaminetetraacetic acid (EDTA) to effectively inhibit the proliferation of E. coli [138]. Secondly, the antibacterial effect can be improved by adding antibacterial metal particles to the material, such as Ag, Cu, and Zn [139, 140]. Finally, researchers can improve the antimicrobial effect of materials by adding antimicrobial drugs or natural antimicrobial reagents, and can adjust the antimicrobial effect according to the dosage of drugs or reagents. Antibacterial drugs include gentamicin, moxifloxacin, ciprofloxacin, etc, and natural antibacterial agents include Centella asiatica, propolis, hinokitiol, etc.

**Fig. 10:**
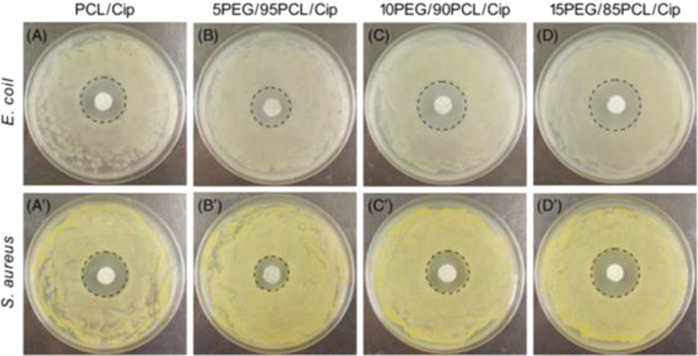
A–D, Bacteria (Escherichia coli) inhibition zone of fiber mats with different component ratios on agar plates; A′–D′, Bacteria (Staphylococcus aureus) inhibition zone of fiber mats with different component ratios on agar plates [141]

Sun et al. [76] created antibacterial silver nanoparticles/chitosan (AgNP/CS) nanocomposites with effective antibacterial activities against Escherichia coli and Staphylococcus aureus strains using in situ synthesis. And the supramolecular assembly of polyethylene glycol-b-polylysine (PEG-b-PLL) and ethylenediaminetetraacetic acid (EDTA) is used to effectively inhibit the proliferation of E. coli [138]. In another study, He et al. [141] also used melt electrospinning to create fiber mats containing various amounts of polyethylene glycol (PEG) polycaprolactone (PCL), and the antibacterial drug ciprofloxacin(Cip), where the ratios of PEG and PCL were 0:100, 5:95, 10:90, and 15:85, respectively, and evaluated the release of ciprofloxacin in experiments. The results showed that the diameter of nanofibers increased and then decreased with increasing PEG content, and the diameters of PCL/Cip, 5PEG/95PCL/Cip, 10PEG/90PCL/Cip, and 15PEG/85PCL/Cip were 123.41 ± 27.92, 41.99 ± 9.06, 136.10 ± 23.82, and 78.72 ± 17.24 μm. In the drug release test, the Cip release of the four composite nanofibers was about 16, 48, 36, and 63% in the first 12 h, respectively. After 168 h of immersion, the Cip release of the four composite nanofibers was about 60, 72, 75, and 90%, respectively. The inhibition was judged by the measurement of the bacterial zone of inhibition, and the mean diameters of the zones of inhibition of the four composite fiber mats for E. coli were 2.49 ± 0.14, 2.18 ± 0.18, 2.64 ± 0.21, and 2.91 ± 0.17 mm, respectively, while for S. aureus, the zones of inhibition of the four composite fibers were 1.92 ± 0.22, 1.86 ± 0.13, 2.32 ± 0.18 and 2.65 ± 0.15 mm. Figure 10 shows the inhibition of Escherichia coli and Staphylococcus aureus by different ratios of nanofibers. However, this method was found to be difficult to avoid the initial burst release of the drug, which leads to short-term antimicrobial effects [137].

### Disease treatment

Traditional disease treatments have drawbacks such as lack of controlled drug release, insufficient drug accumulation in target organs/tissues, or uneven drug distribution in the organism, which can lead to drug toxicity in healthy tissues/cells, resulting in drug side effects, and so on, limiting their use. Polymeric nanofibers are ideal for the delivery of drugs, DNA, and proteins for therapeutic applications because of their high specific surface area/volume ratio, high porosity, and high flexibility [142], at the same time, the development of new polymers, along with other technologies, will result in even better drug-carrying nanofibers that are excellent for drug delivery and provide more effective disease treatment solutions.

Altun et al. [143] used a single-nozzle electrostatic spinning method to prepare hollow particles that could contain amoxicillin; in the meantime, they used polymethylsesimiloxane/chitosan/bovine hydroxyapatite/hexagonal boron nitride blends as raw materials and studied their drug delivery capacity according to the treatment method of osteomyelitis. And Sharma et al. [144] developed a composite nanofiber prepared by electrospinning of polyvinyl alcohol and sodium alginate and loaded with insulin, an antidiabetic drug for the treatment of diabetes. Experiments have verified that the sustained delivery and controlled release of drugs can be achieved by controlling the morphology of the composite nanomaterials. In another study, linalool can be used in biomedicine, cosmetics, antibacterial products, and other industries as natural vegetable oil. Linalool can be used for tumor suppression and treatment of anxiety disorders [145, 146], therefore its application has gotten a lot of attention. Souza et al. [147] investigated the release properties of 10, 15, and 20 wt.% linalool in PLA nanofibrous films prepared by electrospinning and solution blow molding. SEM images showed that the prepared fibers were smooth, with an average diameter of about 200 nm. Drug release experiments showed that the time required to release half of the linalool in solution-jetted nanofibers was 1645 s for 10 wt.% linalool, 411 s for 15 wt.% linalool, 291 s for 20 wt.% linalool, Under the same concentration of linalool, the corresponding times of electrospinning fibers respectively were 575s, 329s, and 76s. Therefore, compared with the PLA nanofibers prepared by electrospinning, the PLA nanofibers prepared by the solution jet method have more durable drug release, which provides a basis for designing a preparation method that better controls the drug release rate.

## Conclusions and future perspectives

The development of a promising DDS is essential to enhancing drug safety, bioavailability, and minimizing negative side effects due to advancements in all biomedical fields as well as the increasing complexity of diagnostic and therapeutic procedures. This review presents a timely and comprehensive summary of polymeric nanofibers for DDS. We first describe the common methods for polymer nanofiber fabrication and then introduce controlled techniques for drug loading into and release from polymer nanofibers. The applications of polymer nanofibers in drug delivery are summarized. In particular, we focus on the relation between the physiochemical properties of polymeric nanofibers and their DDS performance. Overall, this review aims to summarize and discuss the recent advances in polymer nanofibers-based DDS, providing information and guidance for researchers who are interested in the research field. Although polymer nanofibrous materials have obtained exciting research results in DDS, there are still some problems to overcome before their practical applications. The first one is about the toxic problem because most solvents for fabrication of polymer nanofibers materials are toxic. There has been researching on non-toxic solvent systems. For example, Seon-lutz et al. [148] prepared biocompatible insoluble hyaluronic acid nanofibers by using pure water solvent and naproxen, a non-steroidal anti-inflammatory drug, and electrostatic spinning technology. Therefore, suitable materials should be selected and can be naturally degraded after use to promote the development of safe nanofibers. The second concern is about critical factors including air permeability, antibacterial properties, antioxidant effects, sensitivity, and nanofibers recovery rate when to obtain the optimizing conditions. It should be noted that the use of apparatus for large-scale and efficient production of nanofibers membranes is also a difficult problem. Moreover, most studies about polymer nanofibers for DDS are still at the laboratory level. More clinical trials are needed to validate, etc. When the issues discussed above are overcomed, polymer nanofibers for DDS can be applied in wide ranges of applications in biomedical drug delivery, tissue engineering, environmental monitoring, food safety and disease diagnostics.
